# Commentary: Comparison of newer hand-held ultrasound devices for post-dive venous gas emboli quantification to standard echocardiography

**DOI:** 10.3389/fphys.2022.1074436

**Published:** 2023-01-05

**Authors:** Metelkina Asya, Barbaud Axel

**Affiliations:** Azoth Systems, Ollioules, France

**Keywords:** decompression sickness, decompression stress, diving, bubble, Doppler

## 1 Introduction

We read with interest the study by Karimpour et al. (2022) comparing two new portable devices to standard echography on the task of Venous Gas Embolism (VGE) detection in scuba divers. In operating conditions of open water dives, the authors compared portable 2D echograph Butterfly iQ and Doppler ultrasound device O’Dive to a 2D echograph Vivid Q currently used for VGE detection in research. There are however several important aspects of the study that need to be considered.

## 2 Reporting of VGE grades within the scope of use of each device

It is important to ensure the validity of VGE scores by respecting the scope of use of each detector. In contrast to precordial detectors screening for VGE the whole body’s venous return in one measurement, the subclavian detector O’Dive necessitates two measurements, one under each clavicle, for a single VGE evaluation. The O’Dive VGE score is calculated as the highest score within the two. While other research studies followed the user manual and reported correctly the O’Dive VGE scores (Germonpré et al., 2020) (Balestra et al., 2022), the authors of (Karimpour et al., 2022) gave several results outside its scope of use. The tables 5 and 6, sensitivity, specificity and Spearman rho in Section 3.2 are misleading, being based on one-side only subclavian measurements. The VGE grades from O’Dive are correctly reported only in Table 7 (42.7% sensitivity and 86.7% specificity) and in Spearman rho coefficient for maximum grade.

## 3 Dives and monitoring information missing

The authors stated the need to obtain more ultrasonic data in the field conditions as a main motivation of their study. For a field study, the reader would like to have an idea about the range of exposures (depth, duration, gases) being monitored. The inter/intra subject variability in low VGE scores observed after shallow dives can be greater and higher scores from provocative dives could be more stable. Indeed, in (Balestra et al., 2022), the results in 6 divers on provocative dives lead to a very good agreement between grades from O’Dive and grades from a portable echography device. In (Karimpour et al., 2022), 74% of VividQ scores within 141 paired ButterflyiQ/VividQ scores were 0 or 1, suggesting non-provocative diving.

To evaluate monitoring difficulty with each device in operating conditions the reader would need 1) the total number of dives that authors attempted to monitor, 2) the number of dives successfully monitored at least once with at least one device, 3) the number of VGE scores obtained with each device, 4) the number of dives with a full set of measurements for each device. Moreover, it would be pertinent to report if for some divers the monitoring was complicated or impossible due to some physiological characteristics. Unfortunately, the only information about monitored dives and divers provided was the number of subjects enrolled in the study.

## 4 VGE scores included in and excluded from the comparative analysis

The authors excluded from analysis VGE scores from devices when a paired measurement from other device was unavailable. Unfortunately, for O’Dive the authors did not report either number of measurements discarded, or the associated VGE scores. Thanks to the study data communicated by authors, we recovered this information: there were 267 successful measurements with O’Dive and 187 successful measurements with Vivid Q, 40% more O’Dive VGE evaluations were successful compared to Vivid Q. The authors analysed 173 matched pairs of scores from O’Dive and VividQ, thus excluding from analysis 35% of all O’Dive measurements and 7.5% of all Vivid Q measurements. As such a large percentage was excluded from analysis, we summarized in Table 1 the VGE scorings from O’Dive and Vivid Q for both included and excluded data. In particular, we remark that more than half High Bubble Grades (HBG: grades 3 or greater) from O’Dive were excluded from analysis, while it is not the case for Vivid Q.

**TABLE 1:** Number of VGE measurements included in comparative study and excluded measurements from O’Dive and Vivid Q.

O’Dive				**Vivid Q**			
**OD grade**	**All**	**Included**	**Excluded**	**EB grade**	**All**	**Included**	**Excluded**
**0**	195	128	67	**0**	109	98	11
**1**	47	32	15	**1**	21	21	0
**2**	18	10	8	**2**	18	16	2
**3**	3	1	2	**3**	25	25	0
**4**	4	2	2	**4**	12	11	1
				**5**	2	2	0
**Total**	**267**	**173 (65%)**	**94 (35%)**	**Total**	**187**	**173 (93%)**	**14 (7%)**

OD grade: represents VGE scores detected with O’Dive system. EB grade: represents VGE scores detected in Eftedal-Brubakk grading system.

## 5 Equivalence of VGE scores from echographs and doppler detectors

It would be interesting to identify if the difference in VGE scores between Vivid Q and O’Dive comes from the measurement site (subclavian vs. precordial), the technology (Doppler vs. echography) and/or their respective scoring systems. In contrast with classical results (Eftedal and Brubakk, 1997; Brubakk et al., 2001), a recent study (Fichtner et al., 2021) indicates that the EB grades from echography are probably no longer equivalent to Spencer grades from Doppler VGE when evaluation is done at the same measurement site. In (Fichtner et al., 2021), Doppler Spencer grades were systematically lower than echography EB grades, for both precordial and subclavian measurements.

VGE is usually used in research studies to evaluate the DCS risk. Most of historical data relating VGE and DCS risk covers Doppler VGE scored on Kissman-Masurel or Spencer scale (Gardette, 1979) (Vann et al., 1982) (Eatock, 1984) (Sawatzky, 1991) (Hugon et al., 2018), with the two main results for 2D echography EB grades all prior to 2007 (Eftedal et al., 2007) (Doolette, 2016). If the equivalence between scales is lost because of a significant improvement in echograph resolution in VGE detection, the relevance of the additional bubbles detected by echography in DCS risk evaluation should be investigated.

## 6 Conclusion

For medical research, very sensitive VGE detection with a state-of-the-art medical echograph should be preferred when logistics, budget and the availability of a trained technician allow.

Non-etheless, newer devices are opening the way of easy and large-scale studies in the field allowing for wider-ranging investigation of the influence of diverse physiological and external conditions on variability of VGE and decompression risk.

The conclusion (Karimpour et al., 2022) on weak sensitivity of O’Dive compared to Vivid Q based on incorrect (single-side measurements) device usage and unspecified data selection (35% of O’Dive measurements discarded vs. 7% for Vivid Q) in the context of non-provocative diving is unfounded. The conclusion on usability of Butterfly iQ (specificity 85.6%) in studies interested in specificity should also be extended to O’Dive provided its even higher specificity (86.7%).

We also suggest the necessity to reconsider the equivalence of EB vs. Doppler Spencer grades because of the increased sensitivity of echographs (Recommendation 13 of (Mollerlokken et al., 2016)) and to evaluate its importance in DCS risk quantification.
